# Automatic segmentation of ovarian follicles using deep neural network combined with edge information

**DOI:** 10.3389/frph.2022.877216

**Published:** 2022-08-22

**Authors:** Zhong Chen, Changheng Zhang, Zhou Li, Jinkun Yang, He Deng

**Affiliations:** ^1^National Key Laboratory of Science and Technology on Multi-Spectral Information Processing, Key Laboratory for Image Information Processing and Intelligence Control of Education Ministry, School of Artificial Intelligence and Automation, Huazhong University of Science and Technology, Wuhan, China; ^2^Reproductive Medicine Center, Tongji Hospital, Tongji Medical College, Huazhong University of Science and Technology, Wuhan, China; ^3^School of Computer Science and Technology, Wuhan University of Science and Technology, Wuhan, China

**Keywords:** ovarian follicle segmentation, medical image segmentation, deep neural network, deep learning, computer-aided diagnosis

## Abstract

Medical ultrasound imaging plays an important role in computer-aided diagnosis systems. In many cases, it is the preferred method of doctors for diagnosing diseases. Combined with computer vision technology, segmentation of ovarian ultrasound images can help doctors accurately judge diseases, reduce doctors' workload, and improve doctors' work efficiency. However, accurate segmentation of an ovarian ultrasound image is a challenging task. On the one hand, there is a lot of speckle noise in ultrasound images; on the other hand, the edges of objects are blurred in ultrasound images. In order to segment the target accurately, we propose an ovarian follicles segmentation network combined with edge information. By adding an edge detection branch at the end of the network and taking the edge detection results as one of the losses of the network, we can accurately segment the ovarian follicles in an ultrasound image, making the segmentation results finer on the edge. Experiments show that the proposed network improves the segmentation accuracy of ovarian follicles, and that it has advantages over current algorithms.

## Introduction

Ovarian volume, follicle number, and follicle diameter are of great clinical significance (1–4). For example, observing and measuring ovaries and follicles in ultrasound images are the main method to diagnose polycystic ovary syndrome at present. Therefore, accurate segmentation of the ovary and ovarian follicles in ultrasound images has important clinical significance. However, this is also a very challenging work. First, there are a lot of speckle noise in an ultrasonic image (5), which brings difficulties to the segmentation task; second, in ultrasonic image, the edge of the object is fuzzy, so it is challenging to accurately outline the edge of the object; Third, there are changes in the shape and quantity of ovarian follicles. These points make it difficult for us to obtain accurate and detailed segmentation results. Therefore, a method that can automatically and accurately segment the ovary and ovarian follicles in ultrasound images will be particularly meaningful.

For the task of ovarian follicle segmentation, some methods have been proposed in early studies. The methods mainly rely on manually extracted features such as edge (6), texture (7), shape (8), and combination of these features. After extracting features, the methods usually train classifiers to distinguish ovarian follicles from the background. However, although the methods can have a good performance, they rely on accurate feature design to a large extent, and these models often have a high missed detection rate mainly because the representation ability of manually extracted features is limited.

In recent years, many methods based on deep learning have made new progress in the field of medical image segmentation. FCN (9) and Unet (10) are the two most common structures. These methods focus on segmentation based on region but ignore the effect of edges on segmentation. The research of (11–13) shows that advanced features at the object level can be used as clues for edge detection. In turn, edges can also improve the effect of advanced visual tasks such as semantic segmentation. Therefore, we try to use both region and edge information in the task of ovarian follicle segmentation.

In this article, we propose a deep neural network combined with edge information for ovarian follicle segmentation. At the same time, a region and an edge are used as segmentation information, which is also consistent with real life. In the labeling process of ovarian follicles, clinicians first roughly locate ovarian follicles and then accurately outline the edge of ovarian follicles according to local characteristics. Therefore, we suggest that regions and edges are two key information for segmenting ovarian follicles. We first predict the rough area of ovarian follicles and then establish a relationship between the area and the edge. Refer the experimental part of this article for more details.

This article is organized as follows: the first Section Introduction introduces the background of ovarian follicle segmentation and the proposed method. The second Section Related studies introduces related studies on ovarian follicle segmentation. The third Section Method describes the details of our proposed method, including attention mechanism and edge information fusion. The fourth Section Experiment introduces the experimental process, including data set, evaluation index, and experimental results. The fifth Section Results is the conclusion of this article.

## Related studies

In the following, we briefly introduce some studies most related to this article.

The purpose of image semantic segmentation is to automatically classify pixels in an image. Specifically, in medical image segmentation, the purpose is to make the target structure in a medical image clearer. Automatic detection methods based on computer vision tend to be more efficient, cheaper, and more reliable (14), which have important implications in computer-aided diagnosis.

In the traditional medical image segmentation algorithm, (15) an edge detection algorithm based on mathematical morphology as the preprocessing of segmentation is proposed; (16) a shape-based approach to segment medical images is proposed; Gabor filter texture is used to segment medical images (17). These methods have achieved success in some cases, but medical image segmentation is still difficult because of various problems such as low contrast and more noise.

With the development of deep learning, medical image segmentation no longer depends on manually designed features. It has a stronger representation ability and provides better segmentation results. At present, the most popular network is the encoder-decoder structure represented by FCN and Unet, in which the encoder is used to extract image features and the decoder outputs segmentation results from the extracted features. This encoder-decoder has a good structure and can integrate low-level and high-level map image features, and provides an effective solution for medical image segmentation. Unet is expanded by integrating the attention gate in the network (18). The attention gate gives a gating signal to control the important characteristics of pixels in different spatial positions. The image pyramid is applied to the network to fuse multi-scale features using multi-scale information (19). Chen et al. (20) applies the atrous revolution to the deep neural network to effectively expand the receptive field without expanding the parameters to obtain more context information.

The current ultrasonic ovarian image analysis methods can be generally divided into traditional methods and learning-based methods. Traditional methods (6) propose an edge-based method, which takes an image after denoizing and histogram equalization as the segmented input image, uses a Canny operator to obtain the edge, and then carries out feature extraction and classification (7). The target in an ultrasonic image is segmented by texture information. A follicle recognition method is proposed based on extraction of follicle geometric features (8). A Kirsh operator and an optimal threshold method are combined for segmentation (21). The watershed-based method is also applied to this task. Based on the watershed algorithm, follicles are segmented in a semi-automatic way (22). An automatic follicle segmentation method based on watershed and knowledge-based graph search algorithm is proposed (23, 24). It first carries out adaptive filtering on an ultrasonic image then uses the improved marked watershed algorithm to extract the target contour, and finally uses the clustering method to identify the follicle. Regional growth was used for follicle recognition in (25, 26). These studies based on traditional methods have achieved certain results, but they largely depend on fine initialization design, and the characteristics of manual design are also poor in representation ability.

In learning-based methods, (27) a cellular neural network, which is divided into four steps to identify follicles in ultrasound images in which SVM is used to accelerate training, is proposed. In recent years, a method based on deep learning has made new progress in the field of medical ultrasonic image segmentation. FCN (9), Unet (10), Deeplab (20), and Segnet (28) are commonly used networks. Combined with the latest progress of deep learning, it has become a new direction for the ovarian follicle segmentation of ultrasonic images.

## Method

### Combine with edge information

**Figure 1:**
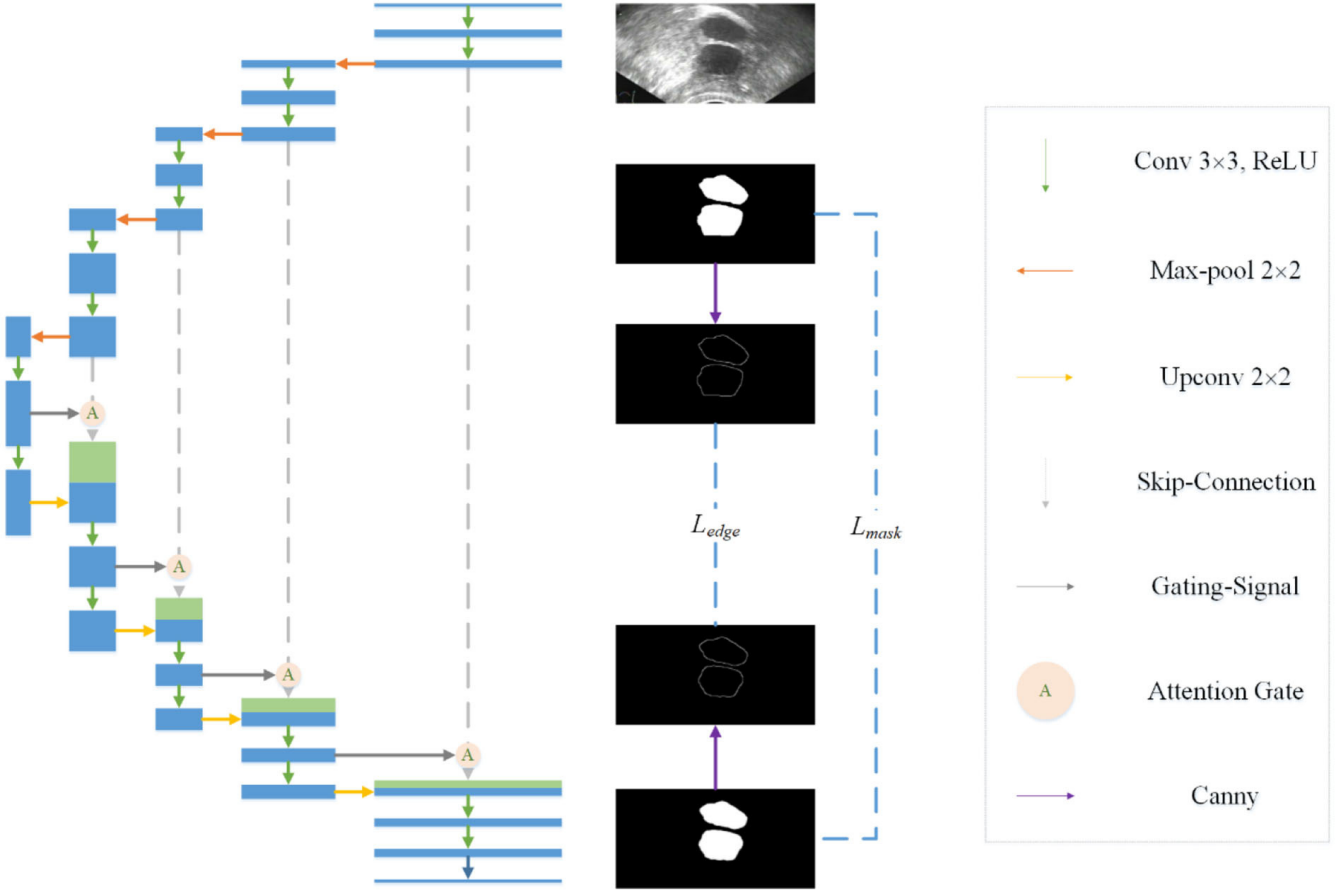
Overall structure of the proposed model.

Observing the segmentation results of Unet, we find that there is a certain gap between the edge of most masks and the real edge of the target, and that even some parts of the target will be lost. Considering the process of visual target segmentation, people do not directly classify the pixels in an image, but they first detect the edge of a target and then fill the closed area surrounded by the edge. The research of (12, 13) also shows that the edge can be used as auxiliary information to improve the effect of segmentation. In order to obtain more precise segmentation results, the edge information of the image is fused into the network. Specifically, by adding branches, edge detection is integrated into the network as a branch, and the edge loss function is added to the joint loss function. The edge loss is generated between the edge detection result and the real edge (see Figure 1).

**Figure 2:**
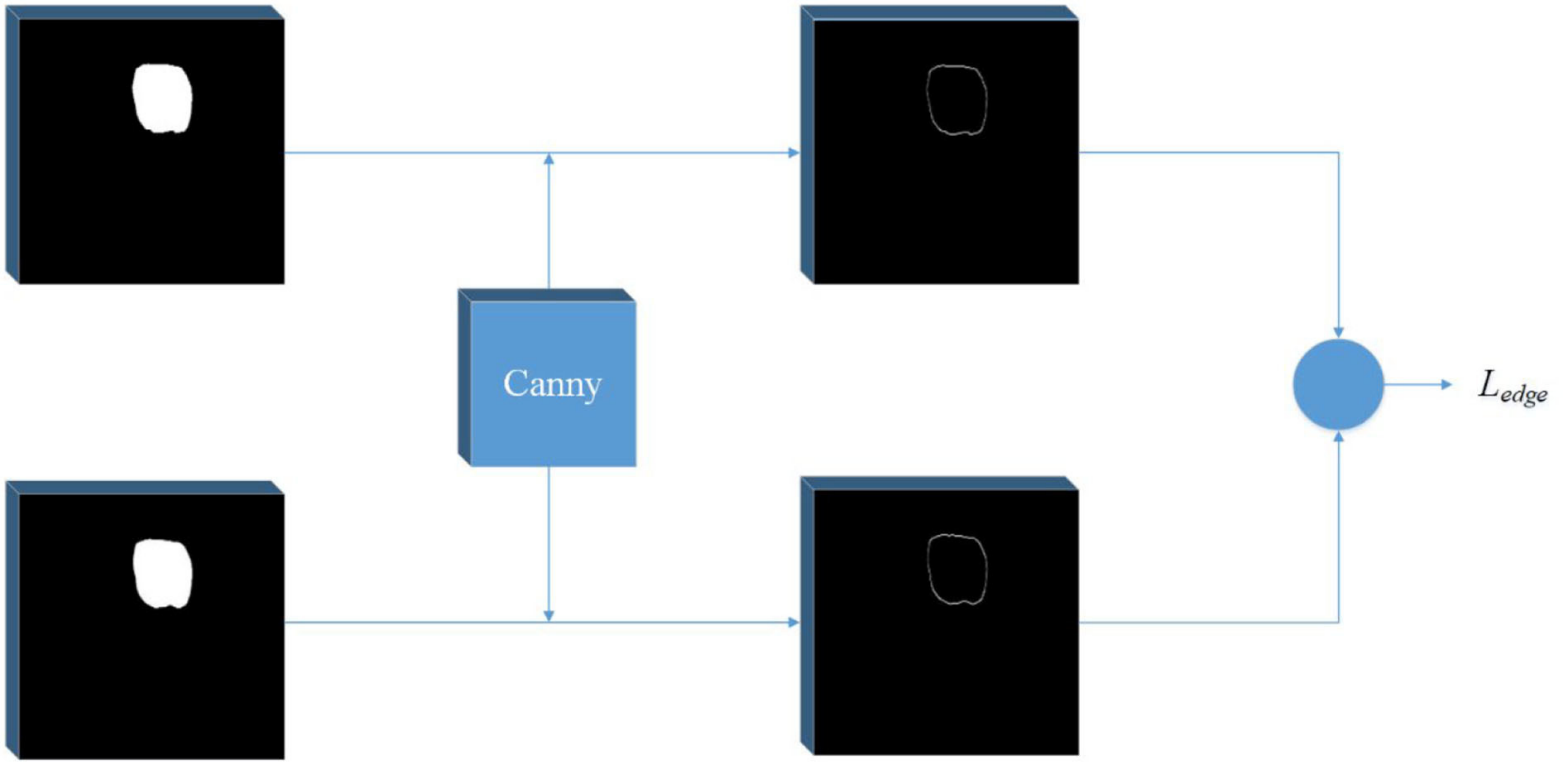
Edge detection network. A Canny operator is used to detect the edge of label image and prediction image at the same time, and then the edge loss can be calculated.


In the field of computer vision, there are many ways of edge detection. We use a Canny operator as an edge detector (see Figure 2). Canny is a popular edge-detection algorithm. For the input image, Sobel horizontal and vertical operators are used to convolute with the input image to obtain the gradient in the horizontal and vertical directions:


(1) $$\begin{array}{l} \begin{array}{c} d_{x}=f{\left(x,y\right)}^{*}Sobel_{x}\left(x,y\right)\\ d_{y}=f{\left(x,y\right)}^{*}Sobel_{y}\left(x,y\right) \end{array} \end{array}$$

Furthermore, the amplitude and angle of the image gradient can be calculated:

(2) $$\begin{array}{l} \begin{array}{c} M\left(x,y\right)=\sqrt{d_{x}^{2}+d_{y}^{2}}\\ \theta_{M}=\arctan\left(d_{x}/d_{y}\right) \end{array} \end{array}$$

Then, the amplitude is suppressed according to the angle, and the final edge result can be obtained by using the double threshold method to detect and connect the edge. In order to get the edge loss, this article constructs a simple network separately. The input of the network is the predicted mask result and the real mask result. The canny operator is used to detect the edge, and then the loss function is calculated.

Considering that there is a serious imbalance between edges and non-edges in ultrasonic images, inspired by (29), we add a balance factor to the cross entropy loss function, which is determined by the proportion of edges and non-pixels in the label. In this way, we determine the edge loss *L*_*edge*_.

The final joint loss function consists of mask loss and edge loss *L*_*edge*_:


(3) $$\begin{array}{l} L_{joint}=\lambda_{1}L_{mask}+\lambda_{2}L_{edge} \end{array}$$

Where λ_1_ and λ_2_ are weight factors.

### Attention mechanism

In ovarian ultrasound images, the ovarian region often accounts for only a small part of the whole image. It is necessary to suppress the irrelevant parts of the image to make the network focus on the ovarian region. A new attention gate model that can automatically learn and focus on targets of different shapes and sizes for computer vision tasks in the medical field is proposed and applied to pancreas CT image segmentation (18).

**Figure 3:**
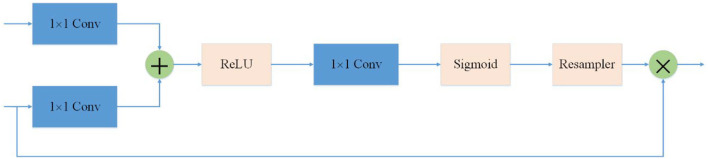
Attention mechanism is used in the skip-connection in the proposed network. As a benefit of using the attention mechanism, the network will focus more on useful region.

In order to suppress irrelevant areas in the input ultrasound image and highlight areas useful for the task, we introduce the attention gate into the proposed network. The attention gate can extract features from the bottom layer, obtain a weighted feature map, and multiply it with abstract features to obtain new features. At the same time, this process can learn how to focus on the target area without additional supervision. This enables us to improve the sensitivity and prediction accuracy of the network without bringing more computational overhead as the cascaded framework extracts the region of interest (see Figure 3).

## Experiment

In this section, we conducted experiments and analyzed the results. Our experiment was conducted under Ubuntu18.04.5 with the NVIDIA RTX3090 GPU model. The deep learning framework is Pytorch1.7.0 with Python3 programming language, and the CUDA version is 11.2.152.

### Dataset

Ultrasonic images were obtained with an Aloka ultrasonic instrument. The original image is a fan-shaped image containing the whole ovary and other regions. In order to avoid the influence of irrelevant regions on segmentation noise, it is necessary to cut the image and extract the region where the ovary is located. Specifically, we choose a window with an appropriate size to ensure that it can contain the complete ovarian region and no other regions as far as possible. In this experiment, the size of the selected window is 512 × 256.

After clipping, 1,050 images of the dataset are obtained. The dataset is divided into training set, verification set, and test set. We label the mask with LabelMe (30) to obtain the accurate mask of ovarian follicles.

### Evaluation metrics

For quantitative analysis of the experiments results, several evaluation metrics are considered, including recall, precision, dice index, and Jaccard similarity. In order to calculate these metrics, True positive (TP), true negative (TN), false positive (FP), and false negative (FN) are used. In this section, GT corresponds to the ground truth and SR to the predicted segmentation mask.

Recall and precision are calculated using the following formulas:


(4) $$\begin{array}{l} Recall=\frac{TP}{TP+FN} \end{array}$$

(5) $$\begin{array}{l} Precision=\frac{TP}{TP+FP} \end{array}$$

Dice index can be defined by the following formula:


(6) $$\begin{array}{l} Dice=\frac{2|GT\cap SR|}{\left|GT|+|SR\right|} \end{array}$$

Jaccard similarity can be defined by the following formula:


(7) $$\begin{array}{l} Jaccard=\frac{\left|GT\cap SR\right|}{\left|GT\cup SR\right|} \end{array}$$

## Results

### Detection results

We evaluated the segmentation performance of our proposed method with the evaluation metrics described in the previous section, including recall, precision, Jaccard, and dice. We used a set of comparative experiments to compare with common segmentation models in the field of medical images, including FCN (9), Segnet (28), and Unet (10). Models are trained for 300 epochs using the Adam optimizer, with a learning rate of 1e-4 and a batch size of 4. All the experiments are performed on the test set, and the results are shown in the following Table 1.

**Table 1:** Comparison of segmentation results.

**Methods**	**Recall**	**Precision**	**Jaccard**	**Dice**
FCN	0.6509	0.7402	0.5298	0.6926
Segnet	0.7396	0.7066	0.5658	0.7227
Unet	0.7884	0.7512	0.6252	0.7694
Ours	0.7773	0.8444	0.6799	0.8095

**Figure 4:**
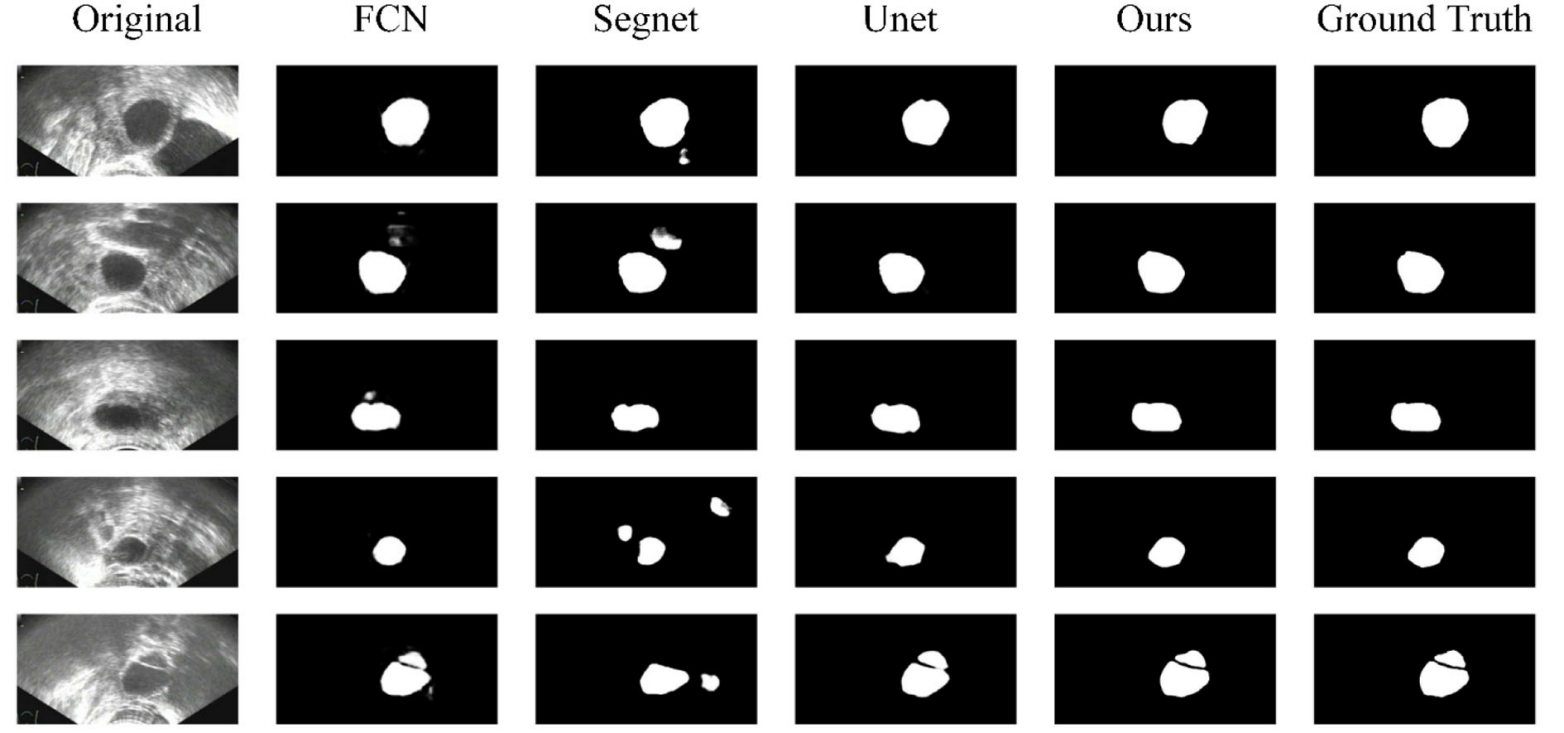
Comparison of segmentation results.

Some segmentation results are shown in Figure 4. From the first column to the last column, the original images, results of FCN, results of Segnet, results of Unet, results of our method, and ground truth are shown, respectively. As can be seen from the results, our method has higher segmentation accuracy than the previous models. The third to fifth rows show the segmentation results under the conditions of strong speckle noise, blurred edge, and quantity change, respectively. In case of strong noise, our model is less likely to be disturbed by noise and can get more refined segmentation results in case of a blurred edge. Furthermore, when faced with more than one object, correct results can be obtained.

To further analyze the influence of each component of our method, we conduct several comparative experiments, and the results are reported. Some of the abbreviations that will be used in this section are EI and AG. EI means edge information, and AM means attention mechanism. This helps us to study the effectiveness of edge information and attention mechanism in our proposed model. The experimental results are given in the following Table 2.

**Table 2:** Comparison of segmentation results using different components.

**Methods**	**Recall**	**Precision**	**Jaccard**	**Dice**
Unet	0.7884	0.7512	0.6252	0.7694
Unet+AM	0.7898	0.7919	0.6541	0.7909
Unet+EI+AM(Ours)	0.7773	0.8444	0.6799	0.8095

**Figure 5:**
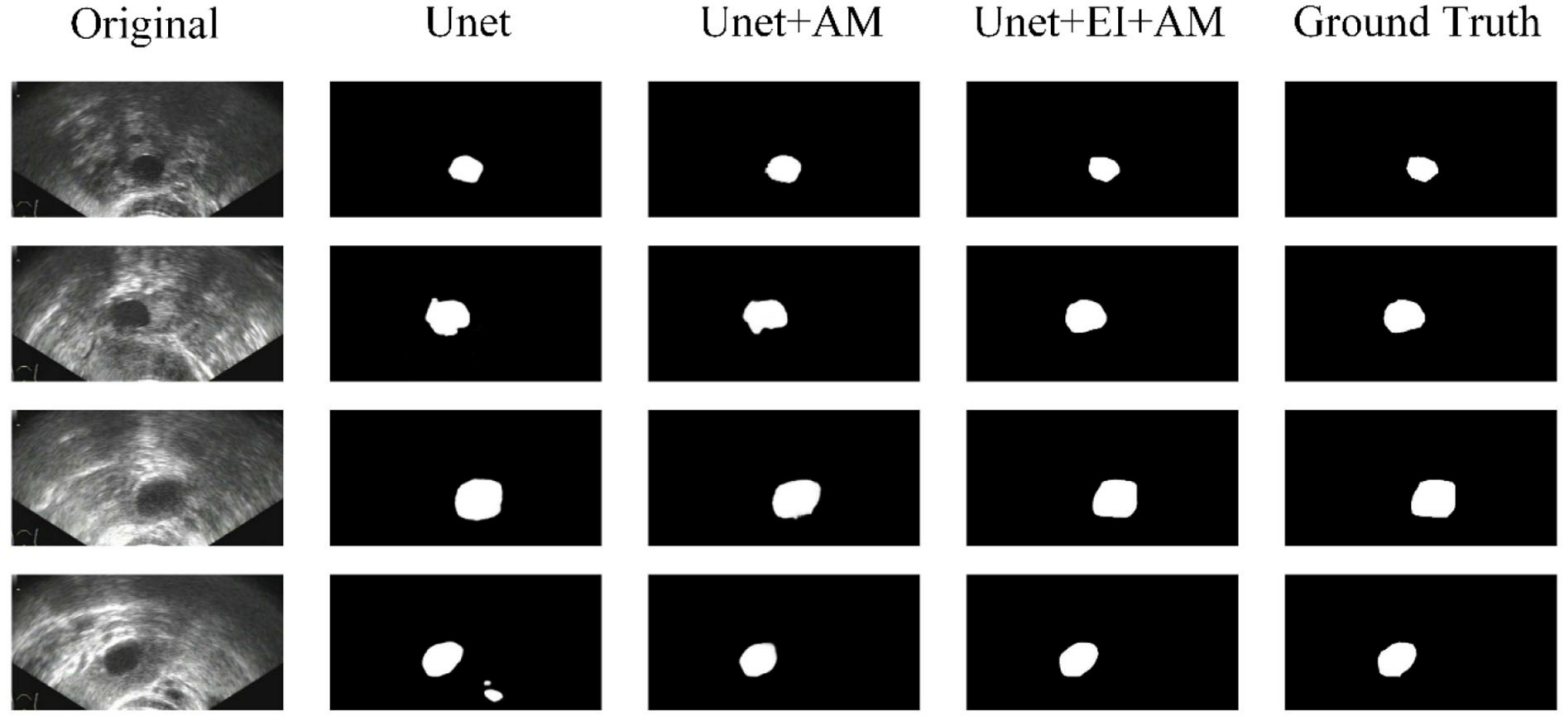
Comparison of segmentation results.

Figure 5 shows some results of the comparison experiment. The first column is the original images, while the last column is the ground truth. The middle three columns are the results of Unet, Unet+AM, and Unet + EI + AM. The attention mechanism could make the network focus more on the useful part, and the edge information is helpful in obtaining finer segmentation results.

## Conclusion

In this article, we proposed an automatic system for automatic segmentation of ovarian follicles in ultrasound images. A deep neural network combined with edge information is used in our system. The attention mechanism used in the skip-connection can make the network focus more on the target region. The combined edge information can improve the network's perception of the edge. At the same time, the joint loss information of mask and edge improves the accuracy of segmentation. The experimental results show that our approach has better performance than some existing models. Our approach can accurately segment ovarian follicles in ultrasound images and obtain a fine edge.

## Data availability statement

The original contributions presented in the study are included in the article/supplementary material, further inquiries can be directed to the corresponding author/s.

## Author contributions

ZC and HD conceived the study. CZ collected data. ZL designed the study. JY analyzed the images. CZ, JY, and ZL wrote the manuscript. ZC and HD critically revised the manuscript. All authors contributed to the article and approved the submitted version.

## Funding

This study was supported by the National Natural Science Foundation of China (62071456), Domestic Satellite Emergency Observation and Information Support Key Technology (B0302), and 13th Five-Year Civil Aerospace Advance Research Project (D040401-w05).

## Conflict of interest

The authors declare that the research was conducted in the absence of any commercial or financial relationships that could be construed as a potential conflict of interest.

## Publisher's note

All claims expressed in this article are solely those of the authors and do not necessarily represent those of their affiliated organizations, or those of the publisher, the editors and the reviewers. Any product that may be evaluated in this article, or claim that may be made by its manufacturer, is not guaranteed or endorsed by the publisher.
